# Where and how does fundamental care fit within seminal nursing theories: A narrative review and synthesis of key nursing concepts

**DOI:** 10.1111/jocn.15420

**Published:** 2020-08-07

**Authors:** Alexandra Mudd, Rebecca Feo, Tiffany Conroy, Alison Kitson

**Affiliations:** ^1^ College of Nursing and Health Sciences Flinders University Adelaide SA Australia; ^2^ Caring Futures Institute Flinders University Adelaide SA Australia; ^3^ International Learning Collaborative

**Keywords:** fundamentals of care, nursing, nursing care, nursing models, nursing theory

## Abstract

**Aims and objectives:**

To elucidate the synergies between fundamental care and seminal nursing theories.

**Background:**

Nursing theories are often criticised for their limited clinical relevance, with the existence of a theory‐to‐practice gap widely acknowledged. Pervasive examples of poor‐quality care, particularly for people's most fundamental needs, raise questions as to whether nursing theories sufficiently prioritise fundamental care. The Fundamentals of Care Framework (hereinafter “the Framework”) represents a valid, comprehensive and evidence‐based description of fundamental care. The Framework captures the complexity and multidimensionality of fundamental care delivery, predicated on the nurse–patient relationship; integration of physical, psychosocial and relational needs; and a supportive context. Despite strong face validity, the Framework's alignment with seminal nursing theories remains unexplored.

**Design:**

Narrative review.

**Method:**

Twenty‐nine seminal nursing theories were included. Categories for analysis were developed inductively and deductively, focusing on the themes of relationship, integration of care, context and the theories’ ease of use. Results are reported in accordance with PRISMA‐ScR guidelines.

**Results:**

Though relationship, integration of care and context and were features shared across a number of nursing theories, no single theory depicts these collectively to the same extent as the Framework. In particular, integration of physical, psychosocial and relational aspects of care was found to be poorly described in the theories.

**Conclusion:**

Failure to account for integration of care means that nursing theories continue to conceptualise fundamental care as a series of discrete tasks. To ensure relevance at the point of care, future nursing theories must accurately reflect the complexities of fundamental care delivery, specifically the need to integrate multiple care needs simultaneously, alongside being straightforward to apply in practice.

**Relevance to clinical practice:**

Bridging the theory‐to‐practice gap requires a nursing discourse that is relevant at the point of care. We provide suggestions for how future nursing theories can bridge this gap.

**What does this paper contribute to the wider global clinical community?**

- Nursing theories have been developed to guide and inform nursing care. However, they have been criticised for their supposed lack of relevance to practising nurses creating a theory‐to‐practice gap.
- This paper investigates whether nursing theories promote fundamental care as a concept that is complex and multidimensional. This is significant as globally there have been high‐profile examples where high‐quality fundamental care is lacking, and attention has turned to exploring the concept of fundamental care as a crucial aspect of the nursing role.
- Results demonstrate that whilst aspects of fundamental care appear to be addressed in existing nursing theories, these aspects are not consistently or sufficiently explicated.
- Most existing theories do not specify whether they were developed in consultation with key stakeholders; the lack of such consultation might further explain the theory‐to‐practice gap.
- To ensure relevance at the point of care, future nursing theories must accurately reflect the complexities of fundamental care delivery by incorporating the nurse–patient relationship, the integration of physical, relational and psychosocial needs and the influence of the care context, whilst focusing on ease of use in clinical practice and education. Areas for future development of the Fundamentals of Care Framework are highlighted.

## 1 INTRODUCTION

Care is a universal aspect of the human experience, yet despite its centrality, there are wide variations in how care is conceptualised, researched and practiced. Care is a broad concept, which is not restricted to, but often epitomised by, nursing. A central tenet of care is fundamental care, which “involves actions on the part of the nurse that respect and focus on a person's essential needs to ensure their physical and psychosocial wellbeing” (Feo, Conroy, et al., 2018, p. 2295). Fundamental care is significant in being the key intersection between caring and nursing. Despite the critical importance of fundamental care, both for patients and the nursing profession, there are numerous examples internationally of deficiencies in fundamental care delivery, leading to poor outcomes and experiences for patients and their families/carers (Francis, 2013; Kalisch, Tschannen, Lee, & Friese, 2011; Royal Commission on Aged Care Quality & Safety, 2019). Understanding why these situations of substandard fundamental care have arisen and how they can be improved requires a coherent shared understanding of fundamental care both as a concept and as daily practice. The Fundamentals of Care Framework, developed by the International Learning Collaborative (ILC), provides such an understanding.

**FIGURE 1:**
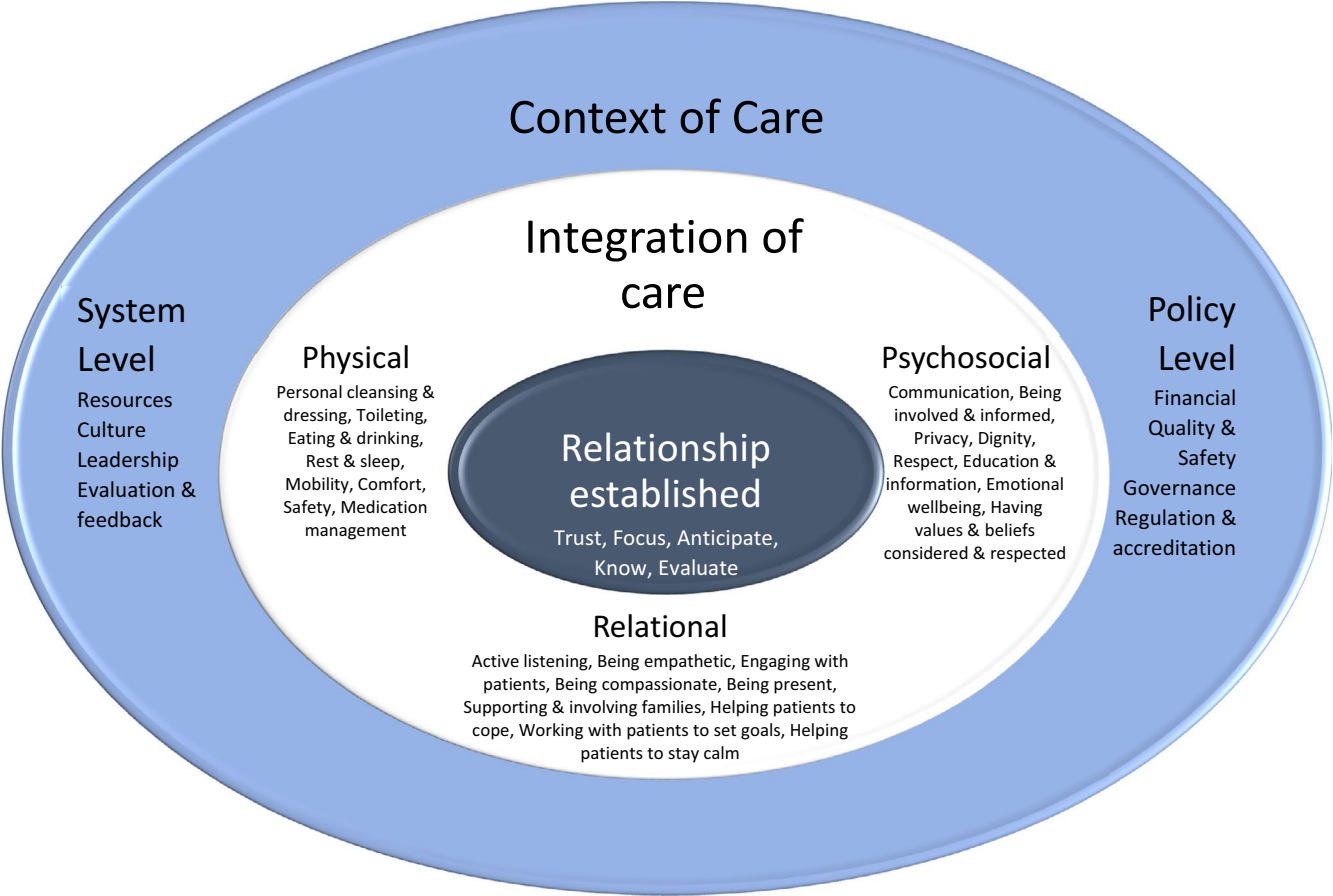
The Fundamentals of Care Framework. Reproduced with permission from the International Learning Collaborative, available electronically at https://intlearningcollab.org/mission/the-fundamentals-of-care/

The ILC is an international network of researchers, clinicians, educators, nursing leaders and consumer representatives, who, through the conduct and application of rigorous research, aim to enhance how fundamental care is delivered and experienced worldwide. One of the inaugural works of the ILC was a meta‐narrative review mapping the terminology pertaining to fundamentals of care. The authors found wide variation across the reviewed documents in a number of areas; the care elements described as comprising fundamental care, the underlying conceptual frameworks involved and the language used (Kitson, Conroy, Wengstrom, Profetto‐Mcgrath, & Robertson‐Malt, 2010). Building upon this knowledge, in 2013, ILC members engaged in a collaborative, participatory codesign approach to identify, using the best available evidence, the key factors for the safe, effective delivery of fundamental care. The resultant conceptual framework, the Fundamentals of Care Framework, posits three key dimensions for high‐quality fundamental care delivery (see Figure 1) (Kitson, Conroy, Kuluski, Locock, & Lyons, 2013):

1. A trusting nurse–patient relationship.
2. The integration of care (a nursing response that simultaneously addresses a persons’ physical, psychosocial and relational needs).
3. A supportive care context.

The Framework's innovation lies in its depiction of fundamental care as multidimensional and multifaceted. In their scoping review on how fundamental aspects of nursing care are defined in the literature, Feo, Kitson, and Conroy (2018) found that aside from the Framework, most literature on fundamental care continued to view fundamental care in terms of a list of discrete (mostly physical) tasks. In contrast, the Framework marked an evolution in thinking from discrete tasks to a complex, integrated activity predicated on establishing and maintaining a trusting relationship between care provider and care recipient (Feo, Kitson, et al., 2018). In addition, the review noted how it was grounded in evidence‐based research and participatory and consensus‐based practice. The scoping review did not find that the Framework was infallible; indeed, it noted that it contained no clear definition of compassion. However, the scoping review was clear that the Framework represents the most comprehensive, evidence‐based definition of fundamental care currently available (Feo, Kitson, et al., 2018). Since its first iteration in 2013, the Framework has been validated through several international studies, including secondary analyses of patient care experiences as well as projects designed to embed the Framework in nursing education (Alderman et al., 2018) and clinical practice (Parr, Bell, & Koziol‐Mclain, 2018). Thus, the Framework offers the current best representation of fundamental care. In addition, rather than being published as a definitive account of fundamental care, the Framework is part of an iterative process and will be refined by group consultation to adapt to changing circumstances. Further details of research and practice relating to the Framework are available at https://intlearningcollab.org.

In view of the innovative ideas expounded by the Framework, the purpose of this review is to examine the synergies between fundamental care (as defined by the Framework) and conceptualisations of nursing as outlined in seminal nursing theories. As a professional group, nursing is identified by its unique body of knowledge and the way that it conceptualises the world. This understanding has been expressed through nursing theories and academic writings on what is nursing and what is nursing's contribution to care. These theories range from Nightingale’s (1863) “do no harm” principle to Henderson's (2004) definition of nursing as assisting the individual to undertake activities to promote recovery and indepleinendence. Nursing theories are seen as crucial for supporting professional autonomy, coherence of purpose and common professional communication and offer a rationale for practice (Chinn, 1983; Colley, 2003; McCrae, 2012). Nursing theories are also a cornerstone of many preregistration courses, where there is an explicit requirement for a theoretical or conceptual framework to underpin curricula (Australian Nursing & Midwifery Accreditation Council, 2012; Chinn, 1999).

Both anecdotally and within the literature, however, there is continued discussion of a theory‐to‐practice gap within nursing (Upton, 1999), to the extent that authors argue the metaphor has become so entrenched as to not require clarification (Gallagher, 2004). Concerns have been raised about the accessibility and clarity of many nursing theories (Scott, 1994). Critics have outlined how, in certain cases, the language used and concepts developed are unclear (McCrae, 2012), or so broadly defined as to lose their meaning (Cash, 1990). The result has been that nurses have disregarded theories as “long winded and irrelevant” (Colley, 2003, p. 33) or as “diversions from intuitive care” (McCrae, 2012, p. 224), with many nurses feeling that theories do not adequately reflect or support their clinical practice (Clarke, 2002). This perception of irrelevancy has led to queries about whether nursing theories are at risk of becoming extinct because they are not relevant at the point of care (Tierney, 1998).

Potentially, the issue is not theories per se but rather the absence of theories that can appropriately guide and explain nursing practice. Kahn and Fawcett (1995) stress that the credibility of nursing theories and models is determined by their social utility. This theme is reiterated by McCrae who states that “the real value of nursing can only be represented by a broad theoretical framework that includes both tested procedures and the humane caring role, and which is operationalised not primarily for research, but for utility” (2012, p. 225).

At present, it is not clear to what extent nursing theories reflect the complexity of fundamental care. This knowledge is important for several reasons; first, for nurses who have adopted a nursing theory to guide their practice, they have a personal responsibility to ensure they are practising in a manner that is up to date with modern practice. As such, adhering to an outdated theory might be detrimental to the care they provide. Second, institutions such as universities and healthcare organisations (particularly the former who as part of their accreditation need to identify their theoretical credentials) must ensure that they are following best practice and providing sufficient resources to meet identified needs. Thus, the theory that one selects will have implications for resource allocation and staff training. Third, it is likely that outside of educational establishments, many nurses and healthcare organisations might not be adopting theories to guide their practice. Without theory to act as a guide, nurses are independently analysing and critically evaluating situations, putting pressure on the individual to personally develop suitable outcomes. In other fields, such as implementation science, there is increasing recognition of the need to adopt a suitable theory within which to frame and guide one's actions (Lynch, Mudge, Knowles, Kitson, Hunter, & Harvey). Before, we cast out theories as irrelevant to nursing, an action that is contrary to developments in other disciplines, we should investigate whether it is their applicability and relevance which is the key issue. We have chosen to investigate this in relation to fundamental care, an area where nursing care internationally has found to be wanting and where recent innovations, such as the Framework, have advanced our knowledge and understanding on the issue. This review will help clinicians, educators and researchers to understand which theories are better suited to promoting high‐quality fundamental care.

## 2 AIMS

The aim of this review was to explore the relationship between seminal nursing theories and fundamental care. Specifically, the purpose is to investigate commonalities between theories and the fundamentals of care to illustrate whether current theories support fundamental care in a meaningful way. Since, it is now understood that fundamental care is complex and multidimensional, rather than a series of discrete tasks, exploring whether existing nursing theories accommodate and/or promote these developments in fundamental care is important. In addition, this is an opportunity to reflect on the Framework, highlighting strengths, deficiencies and areas for further refinement. Since the Framework was developed and continues to be refined using iterative processes, reviews of this kind will help to inform discussions on its future progression. Ultimately, this review will explore whether existing nursing theories fully provide for the high‐quality fundamental care that practising nurses aim to deliver and that nurse educators teach their students about.

## 3 METHODS

From the outset, we recognised that this review would create methodological challenges. First, in seeking to explore the synergies between the fundamental care and nursing theories, we wanted to have a flexible approach to both searching and analysis. Consequently, we did not want to predetermine our categories for analysis prior to the search. Instead, we sought an iterative approach whereby points of analysis would be generated through exploration of both the Framework and the nursing theories. This allowed us to capture the potential wealth of information arising from analysis of the nursing theories. To formalise this iterative process, we adopted an amalgam of narrative review approaches informed by two papers providing guidance on narrative reviews: Baumeister and Leary (1997) and Mays, Pope, and Popay (2005).

Second, the nature of the nursing theories means that what constitutes nursing theory is not always clearly defined. An aspect of this is its historical development, for example, Florence Nightingale is now referred to as a nurse theorist (George, 2011), whereas at the time she was outlining her thoughts and providing advice to carers. Moreover, distinctions between conceptual models, theories and frameworks have not always been clear (Meleis, 2007). In addition, historically nursing theories have been documented in lengthy texts; therefore, key material was likely to be found in books and textbooks rather than journal articles. Consequently, from the outset we were aware that database searching alone would be insufficient and that our primary sources would be nursing textbooks in addition to “snowballing”; investigating references from texts already identified for inclusion (Greenhalgh et al., 2005). By undertaking a narrative review of this type, we acknowledged that the process would be labour and time intensive; however, this is balanced by the richness of the data.

**FIGURE 2:**
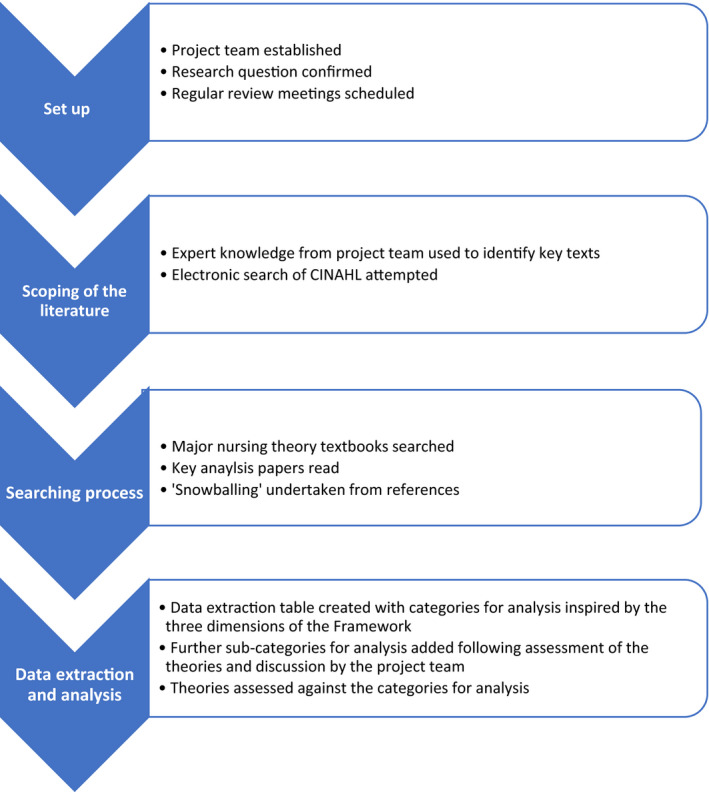
The narrative review process

Our review process involved four stages: set up, scoping of the literature, searching process and data extraction and analysis (see Figure 2).

### 3.1 Set up

Initially, we established our project team and scheduled regular meetings throughout the review process to ensure continual scrutiny and assessment of findings. At this point, we noted the inconsistent use of terminology surrounding frameworks, models and theories as has been routinely noted in the literature (Meleis, 2007). A more nuanced debate on what constitutes a framework, model or theory is beyond the scope of this paper, interested readers can see Meleis (2007). To remain inclusive, we sought to include any conceptual undertaking that informed, underpinned or described nursing care. For the purpose of brevity and to avoid alienating practising nurses through an academic debate on labels, when referring collectively to the conceptual undertakings analysed here, we decided to use the term “nursing theories.” Thereafter, we developed our research question: “*Where and how does fundamental care fit within seminal nursing theories?*”

### 3.2 Scoping of the literature

We undertook an initial scoping search of the literature, drawing on the project team's expert knowledge of seminal nursing texts to identify key nursing theories. In addition, we conducted a preliminary search in December 2019 in CINAHL with the terms nurs* AND theor*, OR framework, OR model. This search retrieved 393,936 results, of which 176,522 were published in the last 10 years. This high volume of results returned from CINAHL demonstrates the extent to which nursing theories continue to feature within the current literature. However, when reading the top results returned by CINAHL, the retrieved articles appeared to make only passing reference to a nursing theory without substantive discussion of its content. Ultimately, database searching was not sufficiently specific or sensitive for our purposes and, accordingly, we heeded Mays et al. (2005, p. 9) guidance to avoid database searching in this scenario.

### 3.3 Searching process

In keeping with the guidance by Mays et al. (2005), our primary aim was for our search to be comprehensive and inclusive. We started by reading *Theory and Nursing: Integrated Knowledge Development* (Chin 1999), which provided an overview of key nursing theories that define the scope, philosophy and general characteristics of nursing. Thereafter, we searched our university library for nursing theory textbooks covering the diversity and breadth of current and historical nursing theories. Three texts describing several theories were identified: *Nursing Theories The Base for Professional Nursing Practice 6*th *edition* (George, 2011), *Nursing Theorists and Their Work 7*th *edition* (Alligood & Marriner‐Tomey, 2010) and *Theoretical Nursing Development and Process 4*th *edition* (Meleis, 2007). In addition, we explored four articles relating to caring in nursing and theory: a discursive piece (Wolf & France, 2017), a meta‐analysis (Swanson, 1999), a meta‐synthesis (Finfgeld‐Connett, 2008) and a comparative analysis (Morse, Bottorff, Neander, & Solberg, 1991). Two of these articles we were aware of prior to commencing the search, and two were retrieved during the CINAHL search. Snowballing took place from references from the textbooks and the articles, and new lines of inquiry were followed where relevant.

#### 3.3.1 Exclusion criteria

We sought to be expansive in our searching and only excluded theories that were restricted to individual health conditions, clinical settings or specialties (thus not overtly addressing fundamental care), alongside those unavailable in English.

### 3.4 Data extraction and analysis

We created a table to facilitate data extraction. Our investigation began initially by focusing on the three dimensions of fundamental care: the nurse–patient relationship, integration of care and context. Upon reading the theories, it became apparent that these dimensions were very broad, and to generate meaningful results, we required more specific points of analysis. Using an iterative process, we read the theories and, as a group, discussed and refined further aspects of the dimensions of fundamental care. Using the nurse–patient relationship dimension as an example, upon preliminary reading of the theories it was clear that some theories highlighted the importance of relationship, whilst others went further and described how a relationship was formed and sustained. We wanted to capture this distinction and so created two separate analysis categories: “highlights importance of relationship” and “describes how relationship is formed and sustained” (see Table 1). In addition, in the theories we noted distinctions pertaining to attributes of the nurse and the respective roles of the nurse and patient, which again expanded our understanding of the nurse–patient relationship. Consequently, for each dimension of the Framework there are several categories of analysis, which were created using this iterative process (see Table 1 for these categories).

**TABLE 1:** Categories and subcategories for analysis

Categories	Demographics/descriptors	Nurse–patient relationship	Integration of care	Context of care	Ease of use
Subcategories	Theory title Author Country of origin Year of first publication Author's description of work (theory, model, framework, philosophy)	Highlights the importance of relationship Describes how the relationship is formed and sustained Internal feelings of the nurse relevant Requires the nurse to be self‐aware Suggestive of nurse to advocate on behalf of patient Nurse and patient share power Nurse supports patient to be in control Patient participates in their care as a respected and autonomous individual	Care plan addresses individual's physical and emotional needs Suggestive of integration of care Explicit discussion of integration of care	Micro level (factors relating to the individual) Meso level (factors relating to the ward/department/area) Macro level (factors relating to broader policy)	Appears easy to use

Also, in the light of the criticisms of nursing theories as being irrelevant (Colley, 2003), we heeded the advice of McCrae (2012) and Kahn and Fawcett (1995) and sought to investigate the concept of social utility. With this in mind and in view of the criticisms of nursing theories relating to language (Scott, 1994), we were keen to assess how easy each theory was to understand and potentially to apply in both education and practice. Thus, we created the category for analysis “ease of use.” In addition, we sought to collect basic demographic and descriptive details for each theory.

Overall, there were five main categories for analysis (demographics/descriptors, nurse–patient relationship, integration of care, context of care and ease of use) and, within these, 21 subcategories. Each theory was assessed against each of the 21 subcategories. To facilitate comparison between the theories, the subcategories for analysis (except for those pertaining to demographic/descriptive details) were each worded to engender a binary response (present or absent). The categories and subcategories for data collection are summarised in Table 1. Results are reported in accordance with the Preferred Reporting Items for Systematic reviews and Meta‐Analyses extension for Scoping Reviews (PRISMA‐ScR) guidelines (Tricco et al., 2018), adapted for this narrative review, see Appendix S1.

## 4 RESULTS

Forty‐five theories were identified via the search. Of these, 16 were excluded from the analysis as they did not overtly address fundamental care or were focused on a particular condition (e.g. Cheryl Beck's Postpartum Depression Theory). Thus, 29 theories were included for analysis. The results are structured in terms of the five main categories for analysis. Thereafter, results are combined to facilitate discussion of multiple factors simultaneously. A list of all included theories is presented in Table 2. Some authors have refined their theories over time. The table provides the name of their most current work, whilst the year provides the first iteration of their ideas. Full details of the analysis for each included theory are presented in a data extraction table see Appendix S2.

**TABLE 2:** List of included theories stating authors name, their theory and year of first iteration of ideas

Author	Theory	Year
Florence Nightingale	Notes on nursing	1859
Hildeguard Peplau	Interpersonal relations in nursing	1952
Lydia Hall	A philosophy of nursing. Care, cure, core theory	1959
Faye Abdellah	Patient centered approaches to nursing	1960
Virgina Henderson	ICN's Basic principles of nursing care	1960
Ida Jean Orlando	Nursing Process Theory	1961
Ernestine Wiedenbach	Clinical nursing a helpful art	1964
Joyce Travelbee	Interpersonal aspects of nursing	1966
Myra Levine	Introduction to Clinical Nursing	1966
Dorothy Johnson	Behavioural system model	1968
Imogene King	A theory for nursing: Systems, concepts, process	1971
Martha Rogers	An introduction to the theoretical basis of nursing ‐ later A science of unitary human beings	1970
Dorothea Orem	Nursing: Concepts or Practice ‐ Self‐care and self‐care deficit theory	1971
Betty Neuman	Neuman systems model	1972
Sister Callista Roy	Introduction to nursing ‐ an adaption model	1976
Josephine Paterson and Loretta Zderad	Humanistic nursing	1976
Jean Watson	Human Caring Science	1979
Nany Roper, Winifred Logan and Alison Tierney	The Roper Logan Tierney model of nursing: based on activities of living	1980
Rosemary Parse	Human becoming school of thought	1981
Patricia Benner	From novice to expert	1984
Madeleine Leininger	Transcultural nursing theory	1985
Margaret Newman	Health as expanding consciousness	1986
Kate Eriksson	Theory of caritative caring	1988
Kari Martinsen	Caring, Nursing and Medicine. Historical philosophical Essays	1989
Kirsten Swanson	Empirical development of a middle range theory of caring	1991
Anne Boykin and Savina Schoenhofer	Nursing as caring	1993
Katharina Kolcaba	Comfort Theory	1994
Sigridur Halldorsdottir	Caring and uncaring encounters in nursing and health care: Developing a theory	1996
Brendan McCormack and Tanya McCance	Person‐centred nursing	2006

### 4.1 Demographics/descriptors

Most included theories (*n* = 23) were developed in the United States, with six in Europe (Finland, Norway, Iceland, the United Kingdom and Ireland). Chronologically, one theory was developed pre‐20th century (Nightingale), and two originate from the 1950s (Peplau and Hall); and seven each from the 1960s (Abdellah, Henderson, Orlando, Wiedenbach, Travelbee, Levine and Johnson), 1970s (King, Rogers, Orem, Neuman, Roy, Paterson & Zderad and Watson) and 1980s (Roper, Logan & Tierney, Parse, Benner, Leininger, Newman, Eriksson and Martinsen). Four were developed in the 1990s (Swanson, Boykin & Schoenhofer, Kolcaba and Halldorsdottir) and one from the 2000s (McCormack & McCance), thus indicating a decline in the generation of theories over time. Predominantly, the theories were generated by a single author. Authors did not always specify which type of theory they had created.

### 4.2 Nurse–patient relationship

Twenty‐five of the 29 theories either directly or implicitly noted the importance of the nurse–patient relationship. For example, Orlando's Nursing Process Theory (1961) directly stressed the importance of relationship in assisting the nurse to interpret the patient's need for assistance. By contrast, Henderson's 14 components of nursing care stressed the value of focusing on the patient and hence are suggestive of, rather than explicit on, the importance of the nurse–patient relationship (1960/2004). Of the 25 theories that either directly or implicitly noted the importance of the relationship, only six directly addressed how a relationship is (or should be) formed or maintained (Peplau, Travelbee, Paterson & Zderad, Benner, Swanson and Halldorsdottir).

Eleven of the 29 theories stated that the internal feelings of the nurse were relevant to their provision of care and relationship with the patient (Orlando, Wiedenbach, Travelbee, Paterson & Zderad, Watson, Eriksson, Martinsen, Swanson, Boykin & Schoenhofer, Halldorsdottir and McCormack & McCance). That is, the theory made reference to the internal motivations and attitudes that nurses should possess when providing care and designated these motivations/attitudes as relevant or central to high‐quality care delivery. This expansive category included the mandate that nurses’ attitude and actions should be authentic. For example, Boykin and Schoenhofer describe how “caring is the intentional and authentic presence of the nurse with another who is recognised as a person living caring and growing in caring” (Boykin, 2001, p. 13). This is an element that the Framework does not explicitly address.

Sixteen theories were found to require the nurse to be self‐aware in their nursing practice (Peplau, Hall, Orlando, Wiedenbach, Travelbee, King, Paterson & Zderad, Watson, Parse, Leininger, Eriksson, Martinsen, Swanson, Boykin & Schoenhofer, Halldorsdottir, McCormack & McCance), which includes all theories published since 1988. An example is found in McCormack and McCance’s (2006) theory on person‐centred care, which describes several prerequisites of the nurse including “knowing self.” The Framework does not expressly specify the need for nurses to be self‐aware in their nursing practice. Within the relationship dimension, the nurse is recommended to evaluate their relationship with their patients, and this may require a certain degree of reflection and self‐awareness by nurses towards their practice, but it is not explicit like some of the nursing theories.

Six theories described how the nurse should advocate on behalf of the patient (Peplau, Hall, Henderson, Orlando, Orem and Halldorsdottir). For example, Peplau described seven nursing roles—stranger, resource, teaching, counselling, surrogate, active leadership and technical expert—where the surrogate role is suggestive of the nurse advocating on behalf of the patient (1952/1988). There was substantial variation in the theories regarding the role of the nurse in creating mutual partnerships with patients. Some theories were suggestive of a powerful role of the nurse in either manipulating the environment (Nightingale) or attempting to interpret patient behaviour (Orlando), whereas others focused on creating mutual partnerships (Peplau, 1988). The Framework is not overt about the need to advocate on behalf of the patient. Within the Framework, there is reference to considering and respecting patients’ values and beliefs and also working with patients to set goals, but there is no reference to advocacy (Halldorsdottir, 1996).

Seventeen of the theories described situations where patients participated in their care as respected and autonomous individuals. However, only 14 of these advocated for a situation where the nurse and the patient shared power. Of these, five went further and indicated that it was the role of the nurse to support the patient to be in control (Peplau, Rogers, Paterson & Zderad, Roper Logan & Tierney, Halldorsdottir). An example is Halldorsdottir's description of nursing as involving “competence in empowering people” (1996, p. 31). Similar to advocacy described above, the Framework specifies the need for nurses to respect patients’ values and beliefs and to support patients but is not explicit about whether nurses should be working towards empowering patients to be in control of their situation.

### 4.3 Integration of care

The concept of integration of care was difficult to assess. Twenty‐two theories described the importance of addressing physical and emotional needs; however, of these, only six suggested integrating these needs (Roper, Logan & Tierney, Benner, Swanson, McCormack & McCance, Martinsen and Halldorsdottir). These six theories are more recent publications (1980, 1984, 1989, 1991, 1996 and 2006), perhaps suggesting a shift in ideas over time. An example of this suggested integration is Martinsen's belief that “*care is a trinity: relational, practical and moral simultaneously*” (Martinsen as cited in Alvsvåg, 2006, p. 175). None of the theories explicitly described integration of care in the manner articulated by the Framework (i.e. simultaneously addressing physical, psychosocial and relational needs). However, conversely, the Framework does not address all of the elements that other theories raise, such as morality (Martinsen as cited in Alvsvåg, 2006) or spirituality (Watson, 2008).

### 4.4 Context of care

The Framework identifies context of care as pertaining to system and policy‐level factors. However, during the review process it became apparent that this description of context in such broad terms, and relating primarily to the immediate context of care, was insufficient to provide insights into the applicability of the theories in practice. Consequently, we recognised that there were potential limitations of the Framework and, for the purpose of our analysis, sought to distinguish between different levels of context to obtain more meaningful results. Given that we are concerned with the applicability of theories to practice, we looked to literature relating to implementing evidence‐based practice in health care (Harvey & Kitson, 2015) and, as a result, formed distinctions between three levels of context: micro (individual factors), meso (ward/department/area factors) and macro (broad policy‐level factors). Using this classification system, 22 theories referred to the context pertaining to the micro (individual) level. Ten theories addressed both micro and meso factors (Wiedenbach, King, Neuman, Roy, Roper, Logan & Tierney, Benner, Leininger, Martinsen, Kolcaba and McCormack & McCance), and only three addressed context at all three levels (Neuman, Roper Logan & Tierney and Leininger). An example is Leininger's Sunrise Model (Leininger & McFarland, 2002), which describes the broad range of factors (technological, religious, kinship, cultural, political and legal, economic and educational) influencing nurses’ worldviews and ultimately care delivery.

### 4.5 Ease of application

Seventeen theories were considered easy to apply in practice (for details, see the data extraction table in Appendix S2). This assessment, made by the project team, considered the relative ease of understanding the theory; whether the theory displayed consistency in its ideas; and whether complex ideas were sufficiently explained using words, graphics or sample documentation. Several theories used graphics and images to help describe the theory, for example Leininger's sunrise model (2002), whilst others such as Roper, Logan and Tierney provided sample documentation to assist implementation. An example of a theory that was deemed difficult to understand and therefore to apply was Parse's human becoming theory. The theory contained complicated language and complex ideas that were not sufficiently or clearly explained for the new user, such as the assumption that “Man is coexisting while coconstituting rhythmical patterns within the environment.” (Parse, 1981, p. 26). In contrast, the Framework was held to be relatively easy to understand, displayed consistency in ideas and explained its elements using definitions and graphics.

### 4.6 Combined results

Fundamental care is premised on the notion that care is multidimensional; thus, we sought to investigate whether any theory sufficiently explored the relationship, integration of care and context of care. Twenty‐five theories highlighted the importance of relationship; however, only six outlined the centrality of the relationship and provided details on how the relationship is formed and maintained (Peplau, Travelbee, Paterson & Zderad, Benner, Swanson and Halldorsdottir). Of these six theories, none explicitly described integration of care (no theory analysed did); however, three of the six were suggestive of integration of care (Benner, Swanson and Halldorsdottir). Of these three, none discussed context at micro, meso and macro levels. Consequently, there was no single theory that emerged as encapsulating the core dimensions of fundamental care. Instead, nursing theories appeared to focus on certain elements of fundamental care whilst not embracing the multidimensionality of such care, specifically the interconnectedness of relationship, integration of care and the care context.

## 5 DISCUSSION

After reviewing the theories against the core dimensions of fundamental care, we identified six major findings that have implications for nursing education and practice:

1. There has been a decline in the number of theories published over time.
2. The importance of relationship is acknowledged in existing theories, yet how this relationship is achieved in practice remains unclear.
3. Existing theories lack a specific and explicit focus on integration of care.
4. The concept of context is poorly developed within both existing theories and the Framework.
5. Ease of use should be a central consideration within nursing theories, but this has frequently been overlooked.
6. A number of learnings have been identified for the Framework.

### 5.1 Decline in the number of theories over time

Our analysis demonstrates there has been a reduction over time in the development of new nursing theories. This could indicate that existing theories are functioning effectively and are fit‐for‐purpose. However, this appears unlikely for two reasons. The first concerns the inconsistencies and deficiencies in fundamental care delivery that continue to challenge healthcare systems globally, demonstrating that clinical practice is not currently guided by a coherent theoretical and conceptual understanding of fundamental care delivery. Second is the existence of a pervasive theory‐to‐practice gap, with many scholars and nurses questioning the relevance of theories to clinical practice and potentially avoiding their use (Colley, 2003; Upton, 1999). It could, of course, be argued that the global deficiencies in fundamental care have arisen because the profession has stopped valuing and using theories to guide their practice. However, the theory–practice gap exists for the very reason that nurses struggle to see the link between nursing theories and the realities of day‐to‐day practice; it does not simply reflect a desire to practice atheoretically. In addition, numerous social, economic and technological changes have also taken place since many of the theories included in this review were generated, a key example being the rise of “person‐centred care,” promoting choice and partnership in care delivery, particularly as it pertains to decision‐making (McCormack, 2004). Without being updated to account for modern conditions, older theories risk becoming outdated and having little to offer in the way of guiding nursing practice.

As the influence of nursing theories on clinical practice has been waning over time, nursing as a profession has embraced, and been encouraged to embrace, other norms to guide practice, such as patient safety and quality frameworks (Hughes, 2008). Our aim is not to critique these endeavours, but to highlight the possibility that nursing priorities are set according to these conceptualisations, which might not always directly align with a focus on fundamental care and in particular the centrality of the nurse–patient relationship. For example, the promotion of patient safety is essential, but there is a distinction between achieving baseline safety and championing optimal fundamental care delivery that enhances the patient's care experience and outcomes. Moreover, many of the clinical manifestations of patient safety and the ways in which this concept has been operationalised relate to discrete aspects of care, such as pre‐operative checklists, which tend to focus on completing a set of disaggregated physical tasks rather than attending to a persons’ fundamental care needs in an integrated manner.

Overall, the decline in publication of nursing theories over time creates the potential for theories to be outdated and lacking in relevance to modern practice, resulting in a conceptual vacuum. If existing theories are not able to address the current lived reality of nursing, then theories become insignificant and there is less imperative to use or produce them. This arguably raises questions as to whether nursing theories are necessary for modern practice or whether nurses can practise safely and effectively without them. However, given that many nurses are currently practicing atheoretically, and we continue to experience deficiencies in fundamental care, and it does not appear that abandoning theories is the answer. Without effective theories, the risk is that nursing could lose its sense of purpose or source of guiding ethics. Nursing theories also continue to be a requirement embedded in nursing curricula and, as such, their existence and use for the present (in education at least) is promoted. This of course could accentuate the theory–practice gap rather than help nurses think conceptually about fundamental care. Consequently, research efforts should focus on scrutinising the content of nursing theories and making suggestions for their improved relevance to practice.

### 5.2 The importance of relationship is acknowledged yet how this relationship is achieved in practice remains unclear

There was strong agreement across most theories and the Framework on the importance of the nurse–patient relationship. However, there was less consistency and explicit articulation around: the issues of patient agency and the role of patients in the relationship; how nurses manage the power differential inherent within the relationship; and how nurses can and should establish, manage and close off the relationship. Indeed, it was found that the Framework was also found not to be explicit in all of these areas. This lack of direction creates challenges for teaching as well as for undertaking and measuring this important dimension of care.

When undertaking the search for this review, we identified several texts that criticised the concept of caring, specifically on the grounds that care disempowers recipients and has an undertone of patriarchy (Rummery & Fine, 2012). In part, these criticisms motivated our investigation in this review into the roles of the nurse and the patient and how the power dynamic is managed. Though many theories aimed for the nurse and patient to share power, few fully acknowledged the inherent power imbalance between nurse and patient and the need for the nurse to actively overcome this by supporting the patient to be in control of their care. Given that patient empowerment is seen globally as a key tenet of healthcare delivery and a process that requires an effective partnership between healthcare professionals and patients (World Health Organisation, 2013), it is imperative that patient empowerment is accurately and explicitly reflected in caring‐based nursing theories and in future iterations of the Framework.

Only six theories fully addressed how to develop and sustain a relationship with a patient (or their family/carers). This absence might suggest that theorists presume nurses already have the requisite skills to develop a relationship or that these skills will come naturally with time or even that these skills are not conducive to instruction. It is also possible that this detail is beyond the scope of some theories and what they are designed to achieve. Theories might be designed at a level of abstraction that does not allow for nor warrant outlining the intricacies of how to create and sustain a relationship. If this is the case, then theories should explicitly signpost to the user their level of abstraction and how the theory is intended to be used, yet many theories included in this review failed to do so. The routine absence of explicit details on how to form and sustain relationships raises questions about whether and to what extent these theories value the relationship and have potentially contributed to the existing theory‐to‐practice gap. Again this might lead certain commentators to query the utility of nursing theories in the nursing discourse. However, if nursing theories continue to underpin nursing curricula globally, then simply dismissing their relevance is not the answer; we must work towards improving the content of theories so that they more readily reflect and predict the realities of nursing practice.

Eleven theories were found to describe how the internal feelings of the nurse were relevant to nurses’ provision of care. Whilst we might expect nurses to act with empathy and to treat patients in a caring manner that respects their dignity, mandating their internal thoughts or feelings is difficult and potentially unrealistic. For instance, several of these theories suggested that nurses’ actions should be authentic and that this was an essential prerequisite for quality care. Whilst we are not advocating for nurses to be inauthentic in their approach with patients, we must question to what extent this authenticity is always required and what impact it might have on nurses. If patients’ needs are appropriately met and they feel well cared for, whether the nurse was “authentic” in their actions and intentions might not be relevant. Indeed, such authenticity might be difficult for nurses to achieve in certain situations, such as when caring for a person with whom they find it difficult to establish a connection or rapport. Requiring specific internal feelings such as authenticity is arguably reminiscent of nursing's origin as a vocation rather than a profession, whereby nurses were required or “called” to attend altruistically to the sick. In practice, nurses that are personally invested in all their patients to this extent run the risk of “burning out” or crossing professional boundaries. Arguably, specifying the internal feelings of the nurse are distinct from the modern concept of nursing as a profession, creating an unrealistic expectation upon nurses and potentially contributing to the perception that theories are irrelevant to clinical practice.

Overall, although the theories noted the importance of the nurse–patient relationship, there were significant gaps in key areas relating to the relationship that would render the theories more readily applicable in practice. Though the Framework highlights the centrality of the relationship and offers guidance on how it is developed and sustained, it does not elaborate explicitly on other aspects such as attributes of the nurse or discuss the role of patient empowerment.

### 5.3 Theories lack a specific and explicit focus on integration of care

There was consistent articulation across the theories of the importance of addressing both physical and emotional needs; however, integration of care was rarely addressed; that is, the need to address simultaneously, and at a minimum, the physical, psychosocial and relational elements of care. There were a limited number of theories that appeared to suggest the importance of integration of care; however, these theories were typically not explicit in describing this integration. For example, Roper, Logan, and Tierney (2001) described their 12 activities of daily living and how these are influenced by biological, psychological, sociocultural, environmental and politico‐economic factors, but did not elaborate on whether or how the actions of the nurse required incorporating these factors during each and every interaction with the patient. In “*From Novice to Expert*” (2001), Benner provided examples where expert nurses appeared able to read situations and respond appropriately and simultaneously to patients’ physical and psychological needs, thereby suggesting integration of care; however, this was not explicitly articulated within the theory.

In each of these theories, true integration of care, that is, bringing together different types of care needs, is not strongly advocated; rather, it is implied. The failure of existing nursing theories to explicitly articulate the importance of integration is arguably a tacit acquiescence of viewing fundamental care as a series of discrete tasks that are basic and which can be completed by anyone, without reflection. The result is that the reader is not enlightened on the mental and physical processes required by the nurse to undertake integration of care and this is significant. First, it suggests that integration of care is accidental rather than planned for. Second, it implies that integration does not warrant detailed explanation or instruction and is therefore not a central tenet of the theories but rather an optional extra. This oversight in relation to integration of care could link to broader concerns regarding the devaluation of care generally and what Colliere refers to as “invisible work” (Colliere, 1986, p. 103). This lack of focus on integration of care also removes the opportunity for experienced nurses who provide such care, to have their experiences described appropriately by nursing theories, leading again to a separation between theory and practice. Without clear articulation, integration remains implicit and the profession loses the opportunity to develop and progress in this area. Though the Framework is explicit about integration, this does not mean that it incorporates all of the elements that are highlighted by other theories. The Framework does refer to considering and respecting patients’ values and beliefs, but explicit reference is not made to spirituality which may be considered an oversight.

### 5.4 The concept of context requires further development

Our initial exploration into context highlighted how the concept, as currently defined by the Framework and relating primarily to the immediate care context, was insufficiently precise to facilitate investigation. Using the three levels of context that we adapted from the implementation science literature (micro, meso and macro), it is arguable that the Framework focuses primarily on the meso. Consequently, we recognise this limitation within the Framework itself.

With regard to the nursing theories, context was consistently identified; however, it was given a variety of meanings, from Parse’s (1992) broad discussion of humans’ interaction with the universe, to more tangible descriptions like Nightingale's (2008) discussion of the health of houses, including elements such as pure air, pure water, efficient drainage, cleanliness and light. Most theories focused on contextual factors relating to the individual (what we designated the micro level), for example factors pertaining to their physical condition, which feature heavily in the examples given by Orlando (1961) and Benner (2001). Theories did not explicitly discuss the role of context in creating safe spaces for the patient, whether this be environmental, physical, emotional/psychosocial or cultural safety, all of which have been identified in the literature as essential aspects of care (Conroy, Feo, Boucaut, Alderman, & Kitson, 2017). Certain theories did highlight the role of families and communities, for example Leininger (1991 p. 22) opines that nursing needs to move beyond its primary focus on the nurse–patient relationship to embrace families, groups, communities and institutions.

Overall, most of the theories appeared to address context in a binary manner, whereby it impacted only the patient or on the nurse. However, health care is infinitely more complicated than this simple understanding. Notarnicola et al. (2017) describe health care as a complex adaptive system and dynamic web, where the individuals, both singularly and in teams, act and react to each other, behaving in a manner that can be unpredictable and which is constantly adapting to the environment. The notion, advanced by some theories, that the nurse interacts solely with the patient and is unfettered by managerial, bureaucratic, socio‐political, economic, regulatory, professional and accreditation related considerations, belies the modern complex reality. Indeed, Feo and Kitson (2016), in their discussion of fundamental care in the acute setting, argue that high‐quality fundamental care delivery is impacted predominantly by contextual factors, chief amongst them the dominance of the biomedical model and the use of managerial incentives that devalue person‐centred care. In her exploration of the modern nursing role, Allen (2014) similarly describes how nurses undertake a large amount of organisational work that distances them from direct patient care. She argues that organisational functions of nursing, such as bed management, are so embedded into the nursing role that nursing academics who fail to consider these functions risk alienating nurses from academic discourse. If nursing theories are to appropriately guide practice, they must therefore take into account these complex contextual factors.

Our exploration of context has demonstrated that both the Framework and existing nursing theories might not sufficiently address context in a manner that is of direct use to practising nurses. Modern nursing is inextricably linked to political influences and therefore requires a theory that equips it with the understanding and appreciation of the complexity of contextual factors. What might help the development of future theories would be for authors to engage with clinicians to fully appreciate the complexity of their role and to understand where and how theories can help to describe and influence practice. It is recommended that further work is undertaken to expand the concept of context as outlined in the Framework and that a participatory collaborative approach adopted.

### 5.5 Ease of use should be a central consideration within nursing theories, but this has frequently been overlooked

Many of the theories analysed were not considered by the research team to be easy to use in practice and education. This conclusion was reached on the grounds that the theories were as follows: difficult to understand, lacked consistency (i.e. concepts described did not appear to be complementary), difficult to be effectively realised or operationalised and lacked assistive documentation to facilitate understanding. Ease of use is essential for theories to be successfully implemented in education and practice. One of the many criticisms of nursing theories has been their purported irrelevancy to practice (Colley, 2003). Benner and Wrubel (1989) propose that one of the reasons for such a disconnect has been that, historically, theories have been generated for curriculum development rather than being shaped by clinical practice, suggesting that there might be a disparity between what is being taught to nursing students and what they experience in clinical settings. Arguably, we need a new understanding of what constitutes nursing theory, moving from purely academic writings of nursing, to a more inclusive approach involving a symbiotic relationship with practice and derived from input from patients, nurses and academics. A core strength of the Framework is that its generation and refinement has been and continues to be inclusive, involving partnership between clinicians, researchers, academics and patient representatives from different healthcare contexts and countries. Furthermore, the iterative nature of the Framework allows it to be adaptive and responsive to the most pressing and current issues affecting our healthcare systems globally. Activities such as this review thus help us to continually refine the Framework ensuring its accuracy and relevance at the point of care.

### 5.6 Learnings for the framework

In undertaking this review, we sought to explore commonalities between theories and the Framework and highlight areas where the Framework could improve. Following the analysis, we propose that there are learnings for each of the three dimensions of the Framework. First, with regard to the relationship, several theories explored nurse attributes, specifically, being self‐aware and authentic in delivery of care which was not explicit in the Framework. Similarly, there is no direct reference within the Framework to nurses acting as patients’ advocates or nurses empowering patients to take control of their situation. Second, in terms of integration of care, certain theories discussed aspects of care that were not directly referred to in the Framework, such as spiritual care. Third, it appears that the context of care is the dimension of the Framework which requires more extensive exploration. At present, the Framework provides a list of relevant factors, but fails to distinguish between those that are micro, meso and macro in nature, and unlike certain theories, does not position the patient within their broader family and community context. Since the Framework is part of an iterative process, these are aspects that can be further discussed, evaluated and refined by future group consultation and research.

### 5.7 Strengths and limitations

A central strength of our search strategy was that it was flexible, allowing us to consult a range of sources and follow new, relevant lines of inquiry. This meant that we reviewed both well‐known theories, such as Peplau's Interpersonal Relations in Nursing (1952/1988), to the more obscure theories, such as Halldorsdottir's Caring and Uncaring Encounters in Nursing and Healthcare (1996). Moreover, our expansive inclusion criteria led to many theories being selected for review. However, this also resulted in a time‐intensive process of reading and analysing to explore the theories in depth and ascertain their unique meaning. Consequently, the searching process and data extraction were undertaken primarily by one member of the project team; however, the whole team met regularly to discuss progress and refine ideas and points for analysis. Finally, our searches were limited to the English language and the results focus heavily on the North American context, where most included theories were developed. A future area of research would be to undertake a search in collaboration with international multilingual colleagues to broaden the scope.

## 6 CONCLUSION

This narrative review explored where and how fundamental care fits within key nursing theories. The findings indicate that each of the core dimensions of the Fundamentals of Care Framework—nurse–patient relationship, integration of care and context of care—can be identified in existing nursing theories however, no single theory fully describes all three dimensions and most theories stop short of explicating these dimensions in sufficient detail. In addition, our review found that many nursing theories were not considered to be easy to use. We also highlighted some areas where the Framework requires further elaboration, discussion and consultation. The fact that many existing nursing theories fail to value and articulate fundamental care and are not easy to understand and use in practice might be contributing to the theory‐to‐practice gap. Given nursing theories underpin nursing curricula, it is imperative that they promote fundamental care and are relevant to clinical practice; this review serves to highlight areas for future development. It is posited that the inclusive and iterative approach of the Framework could be a useful guide for future theory development to ensure relevance at the point of care.

## 7 RELEVANCE TO CLINICAL PRACTICE

This review demonstrates that reliance upon existing nursing theories as a source to guide education and practice might not fully equip students or nurses with the necessary knowledge and skills to easily facilitate high‐quality fundamental care in the clinical setting. To ensure that theories are relevant to clinical practice, this review suggests that key stakeholders, including clinicians and patients, are involved in future theory development and that the key issues highlighted in the review—the nurse–patient relationship, integration of care, context of care and ease of use—feature within any future theory. For clinicians, the iterative and inclusive nature of the Framework offers the opportunity collaborate with other clinicians, researchers, academics and patient representatives to contribute to the evolution of the Framework.

## CONFLICT OF INTEREST

The authors declare no conflict of interest.

## CONTRIBUTIONS

Substantial contribution to conception and design, analysis and interpretation and drafting and revision: AM, RF, TC, AK.

## Supporting information

Appendix S1

Appendix S2
