# Supplementary material for: Where and how does fundamental care fit within seminal nursing theories: A narrative review and synthesis of key nursing concepts
Source: J Clin Nurs. 2020 Aug 7;29(19-20):3652–66. doi: 10.1111/jocn.15420 (PMC7540068; doi:10.1111/jocn.15420)
Supplement: Supplementary file 2 — Appendix S2 [file JOCN-29-3652-s002.docx]

| Demographics / descriptors | | | | | Nurse-patient relationship | | | | | | | | Integration of care | | | Context | | | Ease of application |
| --- | --- | --- | --- | --- | --- | --- | --- | --- | --- | --- | --- | --- | --- | --- | --- | --- | --- | --- | --- |
| Author | Theory | Year | Country | Author's description of work | Highlights importance of relationship | Describes how relationship is formed and sustained | Internal feelings of the nurse relevant | Requires the nurse to be self-aware | Suggestive of nurse to advocate on behalf of patient | Patient participating in their care as a respected and autonomous individual | Nurse and patient share power | Nurse supporting patient to be in control | Care plan addresses individual's physical and emotional needs | Suggestive of integration of care | Explicit discussion of integration of care | Context of care - micro level (factors relating to the individual) | Context of care - meso level (factors relating to the ward / department / area) | Context of care - macro level (broad policy level factors) | Appears easy to use |
| Florence Nightingale | Notes on nursing | 1859 | UK | Notes | ✓ |  |  |  |  |  |  |  | ✓ |  |  | ✓ |  | ✓ | ✓ |
| Hildeguard Peplau | Interpersonal relations in nursing | 1952 | US | Theory | ✓ | ✓ |  | ✓ | ✓ | ✓ | ✓ | ✓ | ✓ |  |  | ✓ |  |  | ✓ |
| Lydia Hall | A philosophy of nursing. Care, cure, core theory | 1959 | US | Philosophy | ✓ |  |  | ✓ | ✓ | ✓ | ✓ |  | ✓ |  |  |  |  |  | ✓ |
| Faye Abdellah | Patient centered approaches to nursing | 1960 | US | Unknown | ✓ |  |  |  |  |  |  |  | ✓ |  |  |  |  |  | ✓ |
| Virgina Henderson | ICN's Basic principles of nursing care | 1960 | US | Unspecified | ✓ |  |  |  | ✓ |  |  |  | ✓ |  |  | ✓ |  | ✓ | ✓ |
| Ida Jean Orlando | Nursing Process Theory | 1961 | US | Theory | ✓ |  | ✓ | ✓ | ✓ | ✓ |  |  | ✓ |  |  | ✓ |  |  | ✓ |
| Ernestine Wiedenbach | Clinical nursing a helpful art | 1964 | US | Unspecified | ✓ |  | ✓ | ✓ |  |  |  |  | ✓ |  |  | ✓ | ✓ |  |  |
| Joyce Travelbee | Interpersonal aspects of nursing | 1966 | US | Theoretical framework | ✓ | ✓ | ✓ | ✓ |  | ✓ | ✓ |  |  |  |  |  |  |  |  |
| Myra Levine | Introduction to Clinical Nursing | 1966 | US | Model | ✓ |  |  |  |  |  |  |  | ✓ |  |  | ✓ |  |  |  |
| Dorothy Johnson | Behavioural system model | 1968 | US | Model | ✓ |  |  |  |  |  |  |  | ✓ |  |  | ✓ |  |  | ✓ |
| Imogene King | A theory for nursing: Systems, concepts, process | 1971 | US | Conceptual system | ✓ |  |  | ✓ |  | ✓ | ✓ |  | ✓ |  |  | ✓ | ✓ |  | ✓ |
| Martha Rogers | An introduction to the theoretical basis of nursing - later A science of unitary human beings | 1970 | US | Unspecified |  |  |  |  |  | ✓ | ✓ | ✓ | ✓ |  |  |  |  |  |  |
| Dorothea Orem | Nursing: Concepts or Practice - Self-care and self-care deficit theory | 1971 | US | Theory | ✓ |  |  |  | ✓ | ✓ | ✓ |  | ✓ |  |  | ✓ |  |  | ✓ |
| Betty Neuman | Neuman systems model | 1972 | US | Model |  |  |  |  |  |  |  |  | ✓ |  |  | ✓ | ✓ | ✓ |  |
| Sister Callista Roy | Introduction to nursing - an adaption model | 1976 | US | Model |  |  |  |  |  | ✓ |  |  | ✓ |  |  | ✓ | ✓ |  |  |
| Josephine Paterson and Loretta Zderad | Humanistic nursing | 1976 | US | Theory | ✓ | ✓ | ✓ | ✓ |  | ✓ | ✓ | ✓ |  |  |  | ✓ |  |  | ✓ |
| Jean Watson | Human Caring Science | 1979 | US | Theory | ✓ |  | ✓ | ✓ |  | ✓ | ✓ |  |  |  |  | ✓ |  |  |  |
| Nany Roper, Winifred Logan and Alison Tierney | The Roper Logan Tierney model of nursing: based on activities of living | 1980 | UK | Model | ✓ |  |  |  |  | ✓ | ✓ | ✓ | ✓ | ✓ |  | ✓ | ✓ | ✓ | ✓ |
| Rosemary Parse | Human becoming school of thought | 1981 | US | Theory |  |  |  | ✓ |  | ✓ | ✓ |  |  |  |  | ✓ |  |  |  |
| Patricia Benner | From novice to expert | 1984 | US | Unspecified | ✓ | ✓ |  |  |  |  |  |  | ✓ | ✓ |  | ✓ | ✓ |  | ✓ |
| Madeleine Leininger | Transcultural nursing theory | 1985 | US | Theory | ✓ |  |  | ✓ |  | ✓ | ✓ |  |  |  |  | ✓ | ✓ | ✓ | ✓ |
| Margaret Newman | Health as expanding consciousness | 1986 | US | Theory | ✓ |  |  |  |  |  |  |  |  |  |  |  |  |  |  |
| Kate Eriksson | Theory of caritative caring | 1988 | Finland | Theory | ✓ |  | ✓ | ✓ |  |  |  |  |  |  |  |  |  |  |  |
| Kari Martinsen | Caring, Nursing and Medicine. Historical philosophical Essays | 1989 | Norway | Philosophy | ✓ |  | ✓ | ✓ |  |  |  |  | ✓ | ✓ |  | ✓ | ✓ |  |  |
| Kirsten Swanson | Empirical development of a middle range theory of caring | 1991 | US | Middle range theory | ✓ | ✓ | ✓ | ✓ |  | ✓ |  |  | ✓ | ✓ |  | ✓ |  |  | ✓ |
| Anne Boykin and Savina Schoenhofer | Nursing as caring | 1993 | US | Model | ✓ |  | ✓ | ✓ |  | ✓ | ✓ |  | ✓ |  |  |  |  |  |  |
| Katharina Kolcaba | Comfort Theory | 1994 | US | Theory | ✓ |  |  |  |  |  |  |  | ✓ |  |  | ✓ | ✓ |  | ✓ |
| Sigridur Halldorsdottir | Caring and uncaring encounters in nursing and health care: Developing a theory | 1996 | Iceland | Theory | ✓ | ✓ | ✓ | ✓ | ✓ | ✓ | ✓ | ✓ | ✓ | ✓ |  | ✓ |  |  | ✓ |
| Brendan McCormack and Tanya McCance | Person-centred nursing | 2006 | Irish | Framework / Mid range theory | ✓ |  | ✓ | ✓ |  | ✓ | ✓ |  | ✓ | ✓ |  | ✓ | ✓ |  | ✓ |
